# Verifying the Breeding Value of A Rare Haplotype of *Chalk7, GS3,* and *Chalk5* to Improve Grain Appearance Quality in Rice

**DOI:** 10.3390/plants11111470

**Published:** 2022-05-30

**Authors:** Dianwen Wang, Jilin Wang, Wenqiang Sun, Xianjin Qiu, Zhiyang Yuan, Sibin Yu

**Affiliations:** 1 National Key Laboratory of Crop Genetic Improvement, Huazhong Agricultural University, Wuhan 430070, China; dianwen1989@126.com (D.W.); wqsun@webmail.hzau.edu.cn (W.S.); yuanzhiyang1989@163.com (Z.Y.); 2 College of Plant Science and Technology, Huazhong Agricultural University, Wuhan 430070, China; wangjilin1982@163.com; 3 Rice Research Institute, Jiangxi Academy of Agricultural Sciences, Nanchang 330200, China; 4 College of Life Science and Technology, Huazhong Agricultural University, Wuhan 430070, China; 5 College of Agriculture, Yangtze University, Jingzhou 434025, China; xjqiu216@yangtzeu.edu.cn

**Keywords:** rice, grain chalkiness, *Chalk7*, *GS3*, *Chalk5*, rare haplotype

## Abstract

Grain quality is a key determinant of commercial value in rice. Efficiently improving grain quality, without compromising grain yield, is a challenge in rice breeding programs. Here we report on the identification and application of a grain quality gene, *Chalk7,* which causes a slender shape and decreases grain chalkiness in rice. Three allele-specific markers for *Chalk7,* and two other grain genes (*GS3* and *Chalk5)* were developed, and used to stack the desirable alleles at these loci. The effects of individual or combined alleles at the loci were evaluated using a set of near-isogenic lines, each containing one to three favorable alleles in a common background of an elite variety. We found that the favorable allele combination of the three loci, which rarely occurs in natural rice germplasm, greatly reduces chalky grains without negatively impacting on grain yield. The data for newly developed allele-specific markers and pre-breeding lines will facilitate the improvement of grain appearance quality in rice.

## 1. Introduction

Rice (*Oryza sativa* L.) is one of the most important staple crops, and provides food for nearly half of the global population. The improvement of grain quality and yield potential is a priority, in order to meet the growing demand of consumers. Grain shape and chalkiness are two important components of grain appearance quality in rice. Grain shape is characterized by grain length, grain width, and the ratio of grain length to width. Chalkiness is usually measured by the percentage of chalky grains, and the area of opaque parts in the endosperm [1]. High grain chalkiness is an undesirable feature in the marketing and consumption of rice, mainly due to its association with inferior rice milling, cooking, and eating quality [1,2]. Slender grains with low chalkiness are preferred by most consumers of rice in southeast Asia [3]. In addition, grain quality and yield seem to be irreconcilable conflicts in rice breeding. Therefore, improving rice varieties with superior grain quality and high yield is a main objective of rice breeding programs.

Both grain shape and grain chalkiness are quantitative traits controlled by quantitative trait loci (QTL), and are largely influenced by environments. Numerous loci were identified for the grain traits by QTL mapping and association analysis in rice (http://www.gramene.org, accessed on 24 April 2017). Dozens of genes conferring grain shape and/or grain chalkiness were cloned in rice, using map-based cloning strategies [4,5,6,7,8,9,10,11,12]. For example, a grain size gene, *GS3,* is highly associated with grain length. The C to A nucleotide substitution in the second exon of *GS3* induces a loss-of-function allele (*gs3*), leading to long grain length [13,14,15,16,17]. *Chalk5* encodes a vacuolar H^+^-translocating pyrophosphatase. Elevating its expression results in increased grain chalkiness. A nucleotide single polymorphism (SNP) in the *Chalk5* promoter (at −721 bp from ATG) is reported to partially account for the expression variation [18]. In this case, due to its tight linkage with *GW5* affecting grain width, unfavorable association of grain width and chalkiness occurred frequently in the rice breeding [5,7]. The grain-related gene *GL7/GW7,* encoding a TONNEAU1-recruiting motif protein, contributes to slender grain and reduced grain chalkiness [19,20]. Intriguingly, this gene does not suggest any association with grain chalkiness by using genome-wide association analysis in rice natural germplasm [21]. Recently, a white-core gene (*WCR1*) was identified to negatively regulate grain chalkiness in rice [22]. A functional A/G variant in the *WCR1* promoter is associated with its expression level. The discovery and characterization of grain-related genes enriched our knowledge on the regulation of grain shape and chalkiness, and facilitates the marker-assisted breeding of high-yield and good quality varieties [6,23,24]. In this context, a large effort is still required to explore multiple beneficial alleles, and develop gene-specific markers for the improvement of grain quality in rice.

Developing functional markers and evaluating breeding values of particular functional alleles are prerequisites for marker-assisted selection (MAS) and genomic breeding in crops. Genome sequence analyses uncovered millions of SNP in rice germplasm, providing the possibility of generating co-dominant markers with high genomic coverage [25,26]. However, there are many challenges in the specificity and availability of use-friendly markers for MAS application [27]. Kompetitive allele-specific PCR (KASP) is a type of high-efficiency, co-dominant marker, combined with a homogeneous, fluorescence-based reporting system [28]. Due to its advantage of high-throughput without the electrophoresis process, over PCR-gel-based markers, the KASP rapidly evolved into a global benchmark technology [29,30,31].

In this study, we verified that the desired alleles of *Chalk7* contribute to both grain shape and grain chalkiness, and developed three KASP markers of *Chalk7* and two other grain genes (*GS3* and *Chalk5*) for the improvement of grain quality. To evaluate the breeding values of the three genes individually, and in combination, we generated a set of near-isogenic lines (NILs) that each contained single or multiple alleles of interest. The favorable allelic combination (haplotype) at the three loci was identified. Due to this optimal haplotype rarely occurring in rice germplasm, we believe that our data, with the availability of allele-specific markers and NIL stocks, will facilitate the improvement of superior grain quality in rice.

## 2. Results

### 2.1. Chalk7 Affects Grain Chalky Rate and Chalkiness Degree in Rice

**Figure 1:**
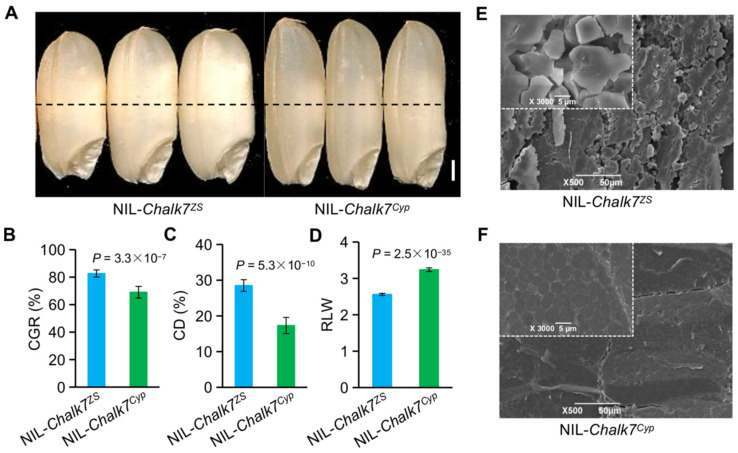
*Chalk7* confers grain chalkiness in rice. (**A**) Grains of NIL-*Chalk7^ZS^* and NIL-*Chalk7^Cyp^*. Scale bar, 1 mm. (**B**–**D**) Differences of NIL-*Chalk7^ZS^* and NIL-*Chalk7^Cyp^* in chalky grain rate (CGR), chalkiness degree (CD), and the ratio of grain length to width (RLW). Data are given as the mean and SE (n = 20). Student’s *t-*test was used to generate *p* values. (**E**,**F**) Scanning electron microscopy images showing starch granule structure of the endosperm of NIL-*Chalk7^ZS^* (**E**) and NIL-*Chalk7^Cyp^* (**F**). The observed section is indicated as the dashed middle bellies of the endosperm in (**A**).

Previously, a major QTL (*qSS7)* for grain shape was delimited into a 23-kb region on chromosome 7 [32]. This region encompasses two candidate genes (*LOC_07g41210* and *LOC_07g41200*). The latter is the gene *GW7*/*GL7,* for long grain length [19,20]. Up-regulation of this gene increases the ratio of grain length to width, and substantially enhances grain appearance quality [19]. Therefore, *GW7*/*GL7* is the candidate gene for grain chalkiness, designated here as *Chalk7*. NIL-*Chalk7^Cyp^*, which carries the *Chalk7* alleles from the Cypress variety (Cyp), has a higher ratio of grain length to width (RLW) than NIL-*Chalk7^ZS^*, harboring *Chalk7^ZS^* from variety Zhenshan97 (ZS) [32]. Besides RLW, lower chalky grain rate (CGR) and chalkiness degree (CD) are observed in NIL-*Chalk7^Cyp^,* relative to NIL-*Chalk7^ZS^* (Figure 1A–D). When examined with scanning electron microscopy, the endosperm of chalky grains of NIL-*Chalk7^ZS^* contains loosely packed starch granules with large air spaces, while those of NIL-*Chalk7^Cyp^* are filled with densely packed granules (Figure 1E,F).

### 2.2. Development of Functional Markers of Chalk7, GS3 and Chalk5

**Figure 2:**
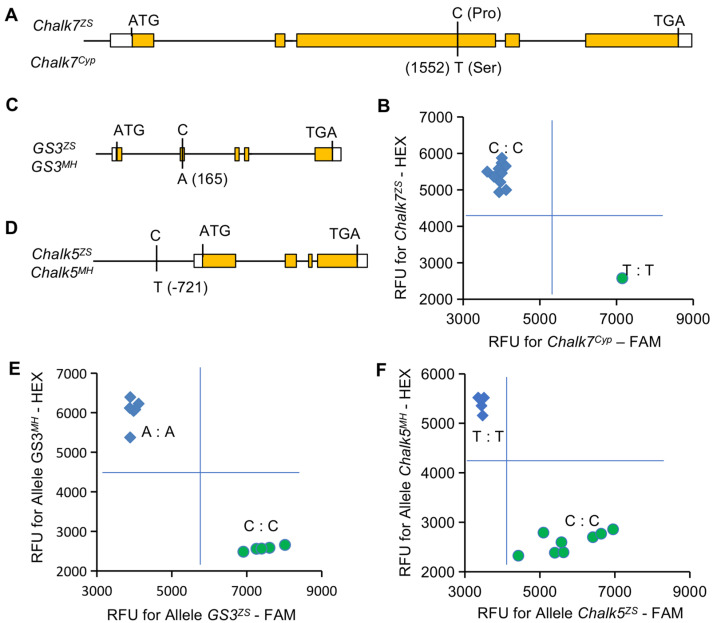
Development of the gene-specific markers for three grain quality genes. (**A**,**C**,**D**) Schematic diagram of the respective gene model showing the position of SNP for the marker development in *Chalk7* (**A**), *GS3* (**C**), and *Chalk5* (**D**). (**B**,**E**,**F**) Genotyping of 12 rice accessions using the markers, K-Chalk7 (**B**), K-GS3 (**E**), and K-Chalk5 (**F**). RFU, relative fluorescence units.

A comparison of sequence variation reveals a non-synonymous SNP (C/T) in the third exon of *Chalk7,* between ZS97 and Cyp (Figure 2A) [32]. Based on this SNP, a allele-specific KASP marker (named K-Chalk7) is developed (Table S1). It distinguishes 12 rice accessions that possess the C or T variants in the gene (Figure 2B, Table S1). *GS3* is the major gene for grain size, and *GS3*^C^^165A^ in the second exon accounts for grain length variation in rice. The gene with SNP^C165^ is a loss-of-function allele (e.g., *GS3^MH^*) that increases grain length (Figure 2C). There is a SNP (C/T) at –721 bp upstream of ATG in the *Chalk5* promoter (Figure 2D), of which *Chalk5^MH^*, with the T variant, reduces grain chalkiness in rice. Based on these SNPs, two KASP markers (K-GS3, K-Chalk5) for corresponding genes are also developed (Table S1). The two markers also accurately recognize the corresponding alleles in the 12 rice accessions (Figure 2E,F, Table S2). To verify the specificity and applicability of the markers, an expanded panel of rice accessions (n = 115) is genotyped (Table S2). The results show that these accessions are classed into two groups with the contrasting alleles by each developed marker.

### 2.3. Validation of Combined Effects of Corresponding Alleles at Three Loci in Rice Germplasm

**Figure 3:**
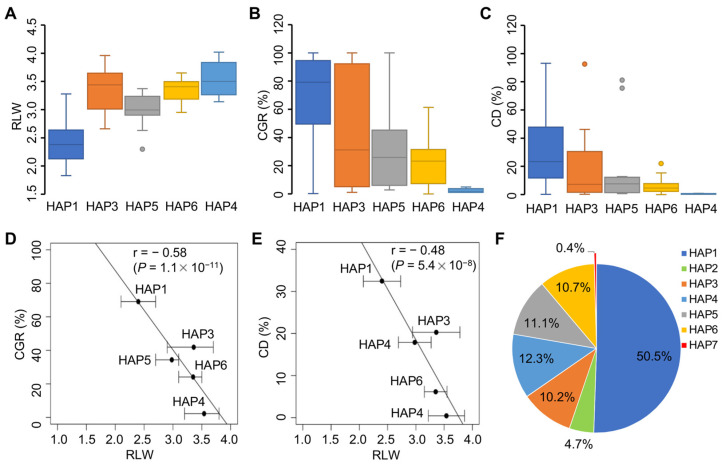
Boxplots showing phenotypic variation of haplotypes assayed by three genic markers in rice germplasm. (**A**) The ratio of grain length to width (RLW); (**B**) chalky grain rate (CGR); and (**C**) chalkiness degree (CD). Box edges represent the upper and lower quantile, with median value shown as bold line in the box. Whiskers represent 1.5 times the quantile of the data. Individuals falling outside the range are shown as open dots. (**D**) Correlation between RLW and CGR among five haplotypes. (**E**) Correlation between RLW and CD. (**F**) Proportions of the haplotypes in a large panel of rice accessions (n = 4694). HAP1, *Chalk7^ZS^/GS3^ZS^/Chalk5^ZS^*; HAP2, *Chalk7^Cyp^/GS3^ZS^/Chalk5^ZS^*; HAP3, *Chalk7^ZS^/GS3^MH^/Chalk5^ZS^*; HAP4, *Chalk7^Cyp^/GS3^MH^/Chalk5^ZS^*; HAP5, *Chalk7^ZS^/GS3^ZS^/Chalk5^MH^*; HAP6, *Chalk7^ZS^/GS3^MH^/Chalk5^MH^*; HAP7, *Chalk7^Cyp^/GS3^MH^/Chalk5^MH^*.

Using three developed markers, the panel of rice accessions (n = 115) is genotyped, and classed into five haplotypes (allelic combinations). Of those haplotypes, HAP1 is the majority (65.2%) of the haplotypes in the panel. ZS97 belongs to HAP1. Thus, HAP1 with the allele combination is designed as *Chalk7^ZS^/GS3^ZS^/Chalk5^ZS^.* It shows a mediate grain shape, and the highest CGR and CD among the five haplotypes (Figure 3A–C). In contrast, HAP4 (*Chalk7^Cyp^/GS3^MH^/Chalk5^ZS^*) is in the minority (4.3%), and reveals a slender shape and the lowest CGR and CD. The grain chalkiness (CGR and CD) is negatively and significantly correlated with RLW among the five haplotypes (Figure 3D,E). These results indicate that the target genes are highly associated with grain shape and chalkiness variation in rice.

In addition, seven haplotypes across the three loci are identified in a large panel of rice germplasms (n = 4694) assayed by the corresponding SNPs in *Chalk7*, *GS3,* and *Chalk5* (http://ricevarmap.ncpgr.cn/hap_net/, accessed on 7 April 2022). HAP1 is consistently the majority (50.5%) of the haplotypes (Figure 3F); HAP2 occurs with less frequency (4.7%), and HAP7 (*Chalk7^Cyp^/GS3^MH^/Chalk5^MH^*) is rarely detected, with only 0.4% in the large panel of rice germplasms.

### 2.4. The Stacking of Chalk7^Cyp^, GS3^MH^ and Chalk5^MH^ Greatly Improved Grain Appearance Quality

**Figure 4:**
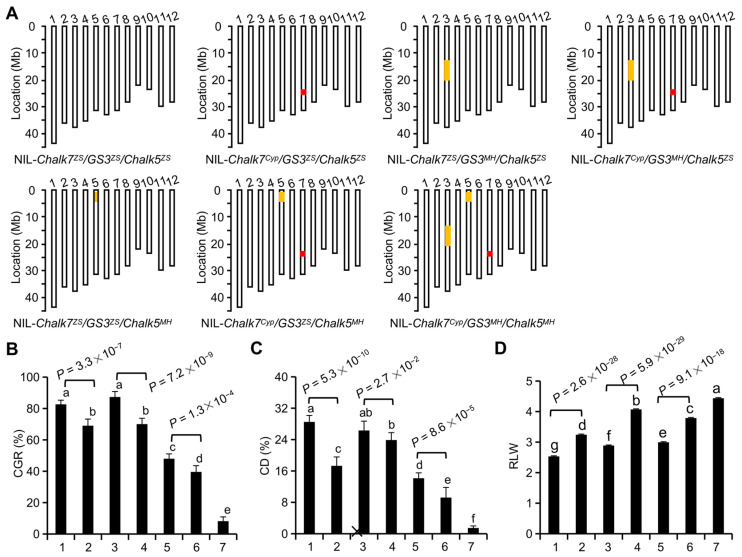
Improving grain quality by pyramiding the alleles, *Chalk7^Cyp^*, *GS3^MH^*, *and Chalk5^MH^.* (**A**) The gene content of the NILs. Chromosomal segments homozygous with respect to MH63 and Cypress are shown as filled yellow and red bars, respectively, and those homozygous with respect to ZS97 are shown as empty bars. (**B**–**D**) Comparative analysis of CGR (**B**), CD (**C**), and RLW (**D**) among the NILs. The number 1 on the x-axial, NIL-*Chalk7^ZS^/GS3^ZS^/Chalk5^ZS^*; 2, NIL-*Chalk7^Cyp^/GS3^ZS^/Chalk5^ZS^*; 3, NIL-*Chalk7^ZS^/GS3^MH^/Chalk5^ZS^*; 4, NIL-*Chalk7^Cyp^/GS3^MH^/Chalk5^ZS^*; 5, NIL-*Chalk7^ZS^/GS3^ZS^/Chalk5^MH^*; 6, NIL-*Chalk7^Cyp^/GS3^ZS^/Chalk5^MH^*; and 7, NIL-*Chalk7^Cyp^/GS3^MH^/Chalk5^MH^*. *p* values for significant differences between paired NILs are based on the student’s *t*-test. Different letters on the bars indicate significant differences among the NILs, based on the Tukey test (*p* < 0.05). Data are given as the mean and SE (n = 16).

To evaluate breeding values of individual or combined alleles at the three loci, a set of NILs, each containing at least one favorable allele in the common background of ZS97, is generated using the marker-assisted backcross scheme (Figure 4A and Figure S1), in which Cypress (carrying *Chalk7^Cyp^*) and Minghui63 (carrying *GS3^MH^* and *Chalk5^MH^*), as the donor parents, are crossed individually with ZS97. A comparison of pairwise NILs containing the contrasting alleles (C*halk7^Cyp^* vs. *Chalk7^ZS^*), even in three diverse genetic backgrounds such as *GS3^ZS^/Chalk5^ZS^*, *GS3^MH^/Chalk5^ZS^*, and *GS3^ZS^/Chalk5^MH^*, reveal that C*halk7^Cyp^* contributes to a significantly reduced CGR and CD and increased RLW, relative to *Chalk7^ZS^* (Figure 4B–D). C*halk7^Cyp^* not only increases grain length by approximately 17.2% relative to *Chalk7^ZS^*, but also decreases CGR and CD by approximately 16.6% and 39.3%, respectively (Table S3). The results indicate that *Chalk7^Cyp^* is a desirable allele, contributing to reduced grain chalkiness and increased grain shape, independent of *GS3* and *Chalk5.*

A comparison of paired NILs, different only in *GS3* region, reveals that *GS3^MH^* improves grain length by approximately 15.2%, and does not significantly change grain chalkiness (Table S3). A similar comparison shows that *Chalk5^MH^* is the major allele, contributing to a decreased CGR by 41.7% and CD by 50.2%, but does not significantly change grain shape.

Regarding the combined two or three favorable alleles, the NIL that pyramids two alleles, *Chalk5^MH^*/*Chalk7^Cyp^*, increases grain length by approximately 19.4%, decreases the CGR by 52.1%, and reduces CD by 67.7%, when compared with ZS97 (Table S3). The NIL carrying *GS3^MH^*/*Chalk7^Cyp^* increases the grain length by approximately 35.6%, and reduces grain chalkiness by approximately 15.2%. The NIL stacking the three alleles (*Chalk7^Cyp^*, *Chalk5^MH^*, and *GS3^MH^*) significantly reduces CGR by 90.1%, and CD by 94.7%.

In addition, there is no significant difference in grain yield among the seven NILs, despite some alterations being observed in panicle length, panicle weight, grain number, and seed setting ratio (Table S3). These results indicate that the combination of three favorable alleles effectively enhances rice grain quality, without compromising grain yield.

## 3. Discussion

### 3.1. Validation of the Chalk7 Effect on Grain Chalkiness

The slender shape and transparency of grains are important characteristics of superior grain quality in rice. In the present study, we find that C*halk7^Cyp^* is a desirable allele using diverse rice germplasm and NILs within the ZS97 background (Figure 3 and Figure 4). It greatly reduces CGR and CD, and increases RLW. Our data support that the *qSS7* and *GL7* regions are associated with grain quality [19,20,32]. Notably, the favorable effects of *Chalk7^Cyp^* on enhanced grain shape and reduced chalkiness are consistent in diverse genetic backgrounds (Figure 4). This suggests that the *Chalk7* effect is independent of *GS3* and *Chalk5.* Many cloned grain-related genes have a pleiotropic effect on agronomic traits, but act in a reverse direction [4,33]. In this case, the major chalkiness gene *Chalk5* is tightly linked to the grain width gene *qSW5* (*GW5*), which results in the unfavorable association of grain width and chalkiness. The use of *Chalk5* for reduced grain chalkiness may require a large population to break this linkage in breeding schemes. Therefore, *Chalk7* will be widely used as a promising gene, and for the good complementary action with other grain genes for the improvement of grain appearance quality in rice.

### 3.2. Stacking Chalk7, GS3 and Chalk5 for Grain Quality Improvement

The development of gene-specific markers is essential to facilitate the precise improvement of target traits by the MAS approach. Previously, many molecular markers of *Chalk7*, *GS3,* or *Chalk5* were developed [18,32,34,35], but most were PCR-gel-based markers, with the disadvantages in high-throughput analysis. In this study, we successfully developed three KASP markers of the three grain quality genes, based on their functional SNPs. The specificity and applicability of the newly developed markers were verified in rice accessions. We found that the majority of rice accessions (50.5%) belongs to the same haplotype as ZS97 (*Chalk7^ZS^/GS3^ZS^/Chalk5^ZS^),* and produce medium grains with high chalkiness (Figure 3), which fail to keep pace with the current demands of grain quality by consumers. As ZS97 was one of the most widely used *indica* maintainer lines in hybrid breeding programs in the past three decades, we attempted to improve its grain quality, and observed that it is difficult to achieve the desired objective using a single gene. Therefore, we used a multi-gene pyramiding strategy. In this context, we found that the optimal allele combination enhances superior appearance quality. NIL-*Chalk7^Cyp^/GS3^MH^/Chalk5^MH^* shows favorable slender grains with RLW of 4.3%, CRG of 8.2%, and CD of 1.5% (Table S3), which meets the appearance criteria for the national standard of high-quality rice. In addition, the fact that the allele combination (haplotype) rarely occurs in a large rice germplasm (Figure 4F) may explain why the gene *Chalk7* is not detected and associated with the grain chalkiness in rice germplasm [21]. These findings of the desired allelic combination and developed functional markers provide an optional strategy and good stocks for the genomic breeding for the improvement of appearance quality in rice.

## 4. Materials and Methods

### 4.1. Plant Materials

NILs were developed using marker-assisted backcross schemes. Briefly, NILs each containing only one introduced region covering a particular gene (*Chalk7^Cyp^*, *GS3^MH^* or *Chalk5^MH^*) in the ZS97 background, were generated by crossing *ZS97* with the donor parents: a tropic *japonica* variety Cypress, and an *indica* variety Minghui63. Then, the NIL*s* containing two or three target genes were generated by pairwise-crossing of the above NILs (Figure S1). During the progression of generations, some progenies were selected to have a background similar to that of ZS97 using 6K gene chip [36], or simple sequence repeat (SSR) markers evenly distributed on rice genomes, as previously described [37].

### 4.2. DNA Extraction and Kompetitive Allele Specific PCR (KASP) Marker Assays

Genomic DNA from fresh young leaves of rice was extracted using the CTAB method [38]. Polymorphic SSR markers were used for MAS. All primers were synthesized at Sangon Biotech (Shanghai, China). For KASP primer design, the functional SNPs for *GS3* and *Chalk5* were targeted, based on previous reports [14,16,18]. The candidate SNP for *Chalk7* was selected based on the sequence variation between *Chalk7*^Cyp^ and C*halk7*^ZS^ (Figure 2A). To ensure the stability and universality of markers, the primer sequences, without any other variations except a particular SNP, were designed, based on the database (http://ricevarmap.ncpgr.cn/, accessed on 4 December 2020). The primer sequences are provided in Table S1. The PCR protocol for KASP was as follows: 5 μL (20–30 ng/μL) DNA, 0.14 μL primer mixture, and 5 μL 2 × KASP Master mixture (KBS-1016-002, LGC, Hoddeston, UK). The cycling regime was as follows: 94 °C for 15 min, 10 touchdown cycles (94 °C for 20 s, touchdown at 61 °C initially, and decreasing by 0.6°C per cycle for 60 s), followed by 28 additional cycles of annealing (95 °C for 20 s, 55 °C for 60 s). The PCR products were detected using the RT-PCR system (CFX-96, BioRad^®^, Hercules, CA, USA).

### 4.3. Trait Measurement

All plant materials were planted in a randomized block design, with two replications, at the experimental station of Huazhong Agricultural University at Wuhan (114°30′ E, 30°60′ N) for phenotype investigation. Each line was grown in a 3 row plot with 10 plants per row, with a spacing of 16.7 cm between plants, and 26.6 cm between rows. The middle eight individuals of each row were harvested for the measurement of agronomic traits, including panicle weight (PW), panicle length (PL), grain number (GN), spikelet number (SN), seed setting ratio (SS), thousand-grain weight (TGW), and yield per plant (YD), as described previously [39]. The seeds were harvested at maturity, and air-dried for 30 days to measure grain-related traits, which included grain length, grain width, the ratio of grain length and width, chalky grain rate, and chalkiness degree. The grain length, grain width, and the ratio of grain length and width were measured with a scanner (5600F, CanoScan, Beijing, China), and digitization was conducted using the software Image J [40]. Chalky grain rate and chalkiness degree were measured using a grain quality analyzer (XLJQ-JMWT12, Beijing, China) [41].

### 4.4. Scanning Electron Microscopy Analysis

For scanning electron microscopy (SEM) analysis, grains were transversely cut with a razor blade. Starch granules were observed with a scanning electron microscope at an accelerating voltage of 12 KV (JSM-6390LV, JEOL, Akishima, Tokyo, Japan). All procedures were performed according to the manufacturer’s protocol [18].

### 4.5. Haplotype Analysis

The functional SNPs of *Chalk7* (vg0724666398), *GS3* (vg0316733441), and *Chalk5* (vg0503340440) were used for haplotype analysis in a panel of 4694 rice accessions. The genomic sequences of these genes in the accession were acquired from RiceVarMap (http://ricevarmap.ncpgr.cn/hap_net/, accessed on 7 April 2022).

## 5. Conclusions

The grain-related gene *Chalk7* was identified as a major contributor to slender grain shape and decreased grain chalkiness in rice. The allele-specific KASP markers for *Chalk7* and two other grain genes (*GS3* and *Chalk5)* were developed for marker-assisted breeding. The effects of the allelic combination at the three loci were evaluated using a series of NILs, within the common background ZS97. The NILs containing the optimal allele combination (*Chalk7^Cyp^/GS3^MH^/Chalk5^MH^*) show a superior grain quality appearance, without compromising grain yield. As this allelic combination is a rare occurrence in rice germplasm, the developed allele-specific markers will be used widely for the improvement of grain quality in the future. The superior allelic combination in the elite genetic background also provides a good startup for genomic breeding, in order to achieve slender grain shape and lower grain chalkiness in rice.

## Data Availability

Data is contained within the article and supplementary material.

## Supplementary Materials

The following supporting information can be downloaded at: https://www.mdpi.com/article/10.3390/plants11111470/s1, Figure S1: Development of near-isogenic lines (NILs) in backcross scheme using marker-assisted selection (MAS); Table S1: Developed KASP primer information; Table S2: Information on rice accessions used in this study; Table S3: Statistical analysis of agronomic traits of different NILs under the ZS97 background.

## References

[1] Fitzgerald M.A., McCouch S.R., Hall R.D. (2009). Not just a grain of rice: The quest for quality. Trends Plant Sci..

[2] Butardo V.M., Sreenivasulu N., Sreenivasulu N. (2019). Improving head rice yield and milling quality: State-of-the-art and future prospects. Rice Grain Quality. Methods in Molecular Biology.

[3] Calingacion M., Laborte A., Nelson A., Resurreccion A., Concepcion J.C., Daygon V.D., Mumm R., Reinke R., Dipti S., Bassinello P.Z. (2014). Diversity of global rice markets and the science required for consumer-targeted rice breeding. PLoS ONE.

[4] Wang S., Wu K., Yuan Q., Liu X., Liu Z., Lin X., Zeng R., Zhu H., Dong G., Qian Q. (2012). Control of grain size, shape and quality by OsSPL16 in rice. Nat. Genet..

[5] Huang R., Jiang L., Zheng J., Wang T., Wang H., Huang Y., Hong Z. (2013). Genetic bases of rice grain shape: So many genes, so little known. Trends Plant Sci..

[6] Zuo J., Li J. (2014). Molecular genetic dissection of quantitative trait loci regulating rice grain size. Annu. Rev. Genet..

[7] Zuo J., Li J. (2014). Molecular dissection of complex agronomic traits of rice: A team effort by Chinese scientists in recent years. Natl. Sci. Rev..

[8] Li N., Li Y. (2016). Signaling pathways of seed size control in plants. Curr. Opin. Plant Biol..

[9] Duan P., Xu J., Zeng D., Zhang B., Geng M., Zhang G., Huang K., Huang L., Xu R., Ge S. (2017). Natural variation in the promoter of GSE5 contributes to grain size diversity in rice. Mol. Plant.

[10] Liu J., Chen J., Zheng X., Wu F., Lin Q., Heng Y., Tian P., Cheng Z., Yu X., Zhou K. (2017). GW5 acts in the brassinosteroid signalling pathway to regulate grain width and weight in rice. Nat. Plants.

[11] Li N., Xu R., Duan P., Li Y. (2018). Control of grain size in rice. Plant Reprod..

[12] Li N., Xu R., Li Y. (2019). Molecular networks of seed size control in plants. Annu. Rev. Plant Biol..

[13] Fan C., Xing Y., Mao H., Lu T., Han B., Xu C., Li X., Zhang Q. (2006). GS3, a major QTL for grain length and weight and minor QTL for grain width and thickness in rice, encodes a putative transmembrane protein. Theor. Appl. Genet..

[14] Fan C., Yu S., Wang C., Xing Y. (2009). A causal C-A mutation in the second exon of GS3 highly associated with rice grain length and validated as a functional marker. Theor. Appl. Genet..

[15] Takano-Kai N., Jiang H., Kubo T., Sweeney M., Matsumoto T., Kanamori H., Padhukasahasram B., Bustamante C., Yoshimura A., Doi K. (2009). Evolutionary history of GS3, a gene conferring grain length in rice. Genetics.

[16] Mao H., Sun S., Yao J., Wang C., Yu S., Xu C., Li X., Zhang Q. (2010). Linking differential domain functions of the GS3 protein to natural variation of grain size in rice. Proc. Natl. Acad. Sci. USA.

[17] Sun S., Wang L., Mao H., Shao L., Li X., Xiao J., Ouyang Y., Zhang Q. (2018). A G-protein pathway determines grain size in rice. Nat. Commun..

[18] Li Y., Fan C., Xing Y., Yun P., Luo L., Yan B., Peng B., Xie W., Wang G., Li X. (2014). Chalk5 encodes a vacuolar H(+)-translocating pyrophosphatase influencing grain chalkiness in rice. Nat. Genet..

[19] Wang S., Li S., Liu Q., Wu K., Zhang J., Wang S., Wang Y., Chen X., Zhang Y., Gao C. (2015). The OsSPL16-GW7 regulatory module determines grain shape and simultaneously improves rice yield and grain quality. Nat. Genet..

[20] Wang Y., Xiong G., Hu J., Jiang L., Yu H., Xu J., Fang Y., Zeng L., Xu E., Xu J. (2015). Copy number variation at the *GL7* locus contributes to grain size diversity in rice. Nat. Genet..

[21] Misra G., Badoni S., Parween S., Singh R., Leung H., Ladejobi O., Mott R., Sreenivasulu N. (2021). Genome-wide association coupled gene to gene interaction studies unveil novel epistatic targets among major effect loci impacting rice grain chalkiness. Plant Biotechnol. J..

[22] Wu B., Yun P., Zhou H., Xia D., Li P., Yao J., Zhou Z., Chen J., Liu R., Cheng S. (2022). Natural variation in WHITE-CORE RATE 1 regulates redox homeostasis in rice endosperm to affect grain quality. Plant Cell.

[23] Zhang L., Ma B., Bian Z., Li X., Zhang C., Liu J., Li Q., Liu Q., He Z. (2020). Grain size selection using novel functional markers targeting 14 genes in rice. Rice.

[24] Pan Y., Chen L., Zhao Y., Guo H., Li J., Rashid M., Lu C., Zhou W., Yang X., Yang X. (2021). Natural variation in *OsMKK3* contributes to grain size and chalkiness in rice. Front. Plant Sci..

[25] Qian Q., Guo L., Smith S.M., Li J. (2016). Breeding high-yield superior quality hybrid super rice by rational design. Natl. Sci. Rev..

[26] Zeng D.L., Tian Z.X., Rao Y.C., Dong G.J., Yang Y.L., Huang L.C., Leng Y.J., Xu J., Sun C., Zhang G.H. (2017). Rational design of high-yield and superior-quality rice. Nat. Plants.

[27] Xu Y., McCouch S.R., Zhang Q. (2005). How can we use genomics to improve cereals with rice as a reference genome?. Plant Mol. Biol..

[28] He C., Holme J., Anthony J., Fleury D., Whitford R. (2014). SNP Genotyping: The KASP Assay. Crop Breeding. Methods in Molecular Biology.

[29] Ertiro B.T., Ogugo V., Worku M., Das B., Olsen M., Labuschagne M., Semagn K. (2015). Comparison of Kompetitive Allele Specific PCR (KASP) and genotyping by sequencing (GBS) for quality control analysis in maize. BMC Genom..

[30] Rasheed A., Wen W., Gao F., Zhai S., Jin H., Liu J., Guo Q., Zhang Y., Dreisigacker S., Xia X. (2016). Development and validation of KASP assays for genes underpinning key economic traits in bread wheat. Theor. Appl. Genet..

[31] Semagn K., Babu R., Hearne S., Olsen M. (2014). Single nucleotide polymorphism genotyping using Kompetitive Allele Specific PCR (KASP): Overview of the technology and its application in crop improvement. Mol. Breed..

[32] Qiu X., Gong R., Tan Y., Yu S. (2012). Mapping and characterization of the major quantitative trait locus qSS7 associated with increased length and decreased width of rice seeds. Theor. Appl. Genet..

[33] Choi B., Kim Y., Markkandan K., Koo Y., Song J., Seo H. (2018). GW2 functions as an E3 ubiquitin ligase for rice expansin-like 1. Int. J. Mol. Sci..

[34] Ramkumar G., Sivaranjani A.K.P., Pandey M.K., Sakthivel K., Shobha Rani N., Sudarshan I., Prasad G.S.V., Neeraja C.N., Sundaram R.M., Viraktamath B.C. (2010). Development of a PCR-based SNP marker system for effective selection of kernel length and kernel elongation in rice. Mol. Breed..

[35] Wang C., Chen S., Yu S. (2011). Functional markers developed from multiple loci in GS3 for fine marker-assisted selection of grain length in rice. Theor. Appl. Genet..

[36] Yu H., Xie W., Li J., Zhou F., Zhang Q. (2014). A whole-genome SNP array (RICE6K) for genomic breeding in rice. Plant Biotechnol. J..

[37] Mccouch S.R., Teytelman L., Xu Y., Lobos K.B., Clare K., Walton M., Fu B., Maghirang R., Li Z., Xing Y. (2002). Development and mapping of 2240 new SSR markers for rice (*Oryza sativa* L.). DNA Res..

[38] Murray M.G., Thompson W.F. (1980). Rapid isolation of high molecular weight plant DNA. Nucleic Acids Res..

[39] Wang P., Zhou G., Yu H., Yu S. (2011). Fine mapping a major QTL for flag leaf size and yield-related traits in rice. Theor. Appl. Genet..

[40] Rha E.Y., Kim J.M., Yoo G. (2015). Volume measurement of various tissues using the image J software. J. Craniofac. Surg..

[41] Sun W., Zhou Q., Yao Y., Qiu X., Xie K., Yu S. (2015). Identification of genomic regions and the isoamylase gene for reduced grain chalkiness in rice. PLoS ONE.

