# Supplementary material for: Verifying the Breeding Value of A Rare Haplotype of Chalk7, GS3, and Chalk5 to Improve Grain Appearance Quality in Rice
Source: Plants (Basel). 2022 May 30;11(11):1470. doi: 10.3390/plants11111470 (PMC9182975; doi:10.3390/plants11111470)
Supplement: Supplementary file 1 [file plants-11-01470-s001.zip › Figure S1.pdf]

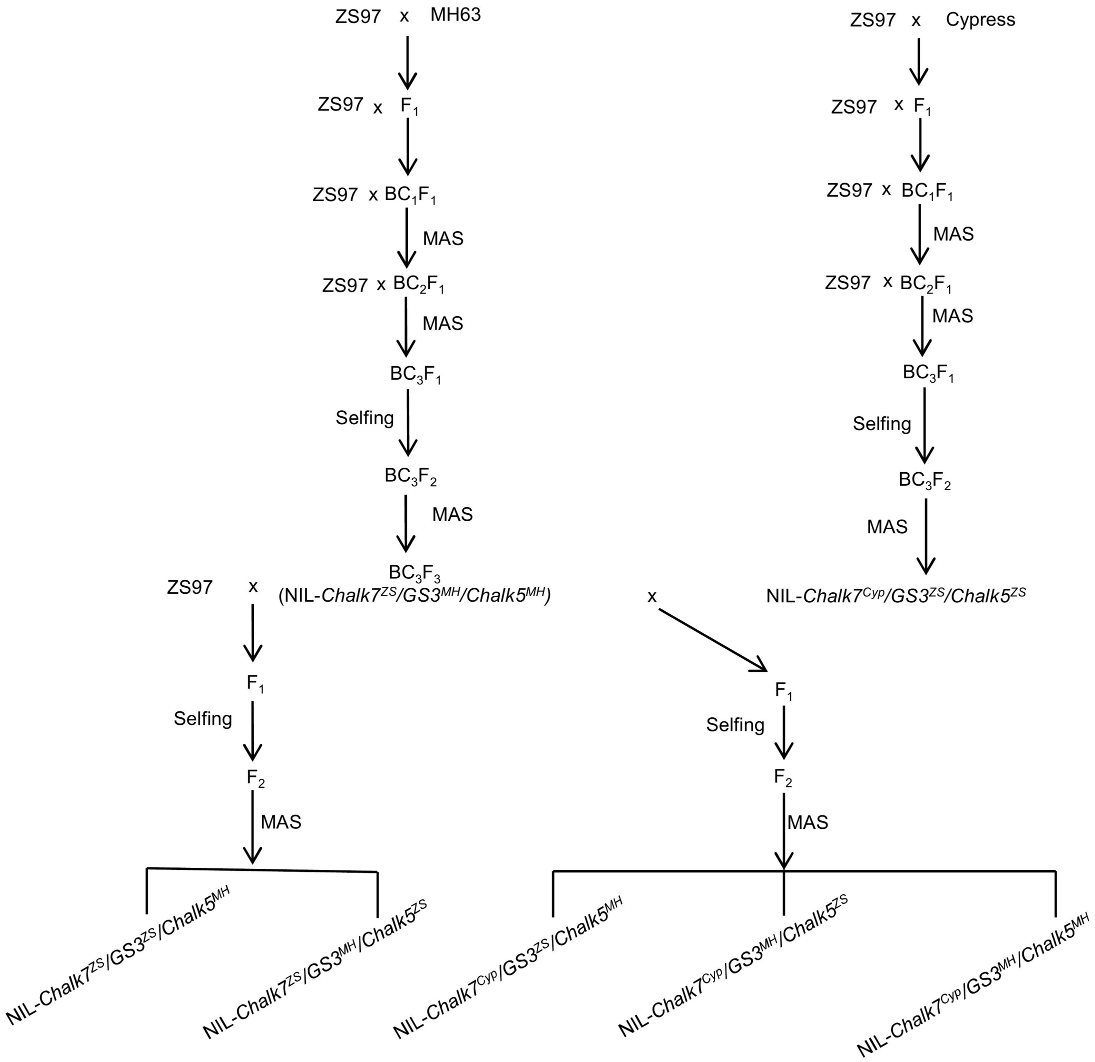

Figure S1: Development of near-isogenic lines (NILs) in backcross scheme using molecular assisted selection (MAS). The favorable allele *Chalk7*^{Cyp} comes from Cypress and *GS3*^{MH} and *Chalk5*^{MH} from Minghui63 (MH).
